# Chromosomal genome and population genetic analyses to reveal genetic architecture, breeding history and genes related to cadmium accumulation in *Lentinula edodes*

**DOI:** 10.1186/s12864-022-08325-x

**Published:** 2022-02-10

**Authors:** Hailong Yu, Lujun Zhang, Xiaodong Shang, Bing Peng, Yu Li, Shijun Xiao, Qi Tan, Yongping Fu

**Affiliations:** 1 Institute of Edible Fungi, Shanghai Academy of Agricultural Sciences, National Engineering Research Center of Edible Fungi, Shanghai, 201403 China; 2 Internationally Cooperative Research Center of China for New Germplasm Breading of Edible Mushroom, Jilin Agricultural University, Changchun, 130018 China; 3 Jiaxing Key Laboratory for New Germplasm Breeding of Economic Mycology, Jiaxing, 314000 China

**Keywords:** *Lentinula edodes*, Germplasm evaluation, Genome, Population genetic analysis, GWAS, Cd accumulation

## Abstract

**Background:**

*Lentinula edodes* (Berk.) is the second most productive mushroom in the world. It contains compounds effective for antiviral, antitumor, antioxidant and immune regulation. Although genomes have previously been reported for this species, a high-quality chromosome-level reference for *L. edodes* is unavailable. This hinders detailed investigation of population genetics, breeding history of strains and genes related to environmental stress responses.

**Results:**

A high-quality chromosome-level genome was constructed. We separated a monokaryon from protoplasts of the commercial *L. edodes* strain L808 and assembled the genome of *L. edodes* using PacBio long-read and Illumina short-read sequencing, along with the high-throughput chromatin conformation capture (Hi-C) technique. We assembled a 45.87 Mb genome, and 99% of the sequences were anchored onto 10 chromosomes. The contig and scaffold N50 length were 2.17 and 4.94 Mb, respectively. Over 96% of the complete Benchmarking Universal Single-Copy Orthologs (BUSCO) were identified, and 9853 protein-coding genes were predicted. We performed population genome resequencing using 34 wild strains and 65 commercial cultivars of *L. edodes* originating from China, Japan, the United States and Australia. Based on whole-genome variants, we showed substantial differences in the Chinese wild population, which divided into different branches according to the main areas of their geographical distribution. We also determined the breeding history of *L. edodes* at the molecular level, and demonstrated that the cultivated strains in China mainly originated from wild strains from China and Northeast Asia. Phenotypic analysis showed that 99 strains exhibited differences on the Cd accumulation. Three significant loci in the of *L. edodes* genome were identified using the genome-wide association study (GWAS) of Cd accumulation traits. Functional genes associated with Cd accumulation traits were related to DNA ligase and aminoacyl tRNA synthetase, indicating that DNA damage repair and in vivo protein translation may be responses to Cd stress.

**Conclusions:**

A high-quality chromosome-level genome and population genetic data of *L. edodes* provide genetic resources for functional genomic, evolutionary and artificial breeding studies for *L. edodes*.

**Supplementary Information:**

The online version contains supplementary material available at 10.1186/s12864-022-08325-x.

## Background

*Lentinula edodes* (Berk.), commonly known as “Shiitake” and “Xianggu”, is an edible and medicinal mushroom that cultivated worldwide [1, 2]. *L. edodes* contains compounds effective in antiviral, antitumor, antioxidant and immune treatment [3–5]. *L. edodes* has been cultivated in China for more than 800 years [6], and production totaled 11.16 million tons in 2019. There was a 30-fold production increase between 1978 and 2019 [7]. *L. edodes* can be cultivated using agricultural and forest waste, enabling waste biotransformation [8, 9]. *L. edodes* can absorb cadmium (Cd), a heavy metal pollutant in cultivation substrates [10]. Chinese National Standard stipulates that the maximum value of Cd in fresh *L. edodes* should not exceed 0.5 mg/kg. A high Cd level in the fruiting bodies can be toxic to humans [11] and may decrease the intelligence of children [12]. Cd can accumulate over the lifetime of an organism [13], so even small amounts of Cd can eventually affect human health. Therefore, the quality and safety of *L. edodes* on Cd accumulation is a public concern. However, few studies have evaluated the level of Cd accumulation in *L. edodes* and determined potential key genes contributing to Cd accumulation.

High-throughput sequencing technologies have been used to study the genomic differences and transcription levels in edible mushrooms [14–17]. These data provide insights into the differences between germplasms and the causes of diversity. There are seven de novo sequences of *L. edodes* available from the NCBI database. Among them, six were assembled by next-generation sequencing technology. The scaffold numbers all exceed 300 [18–20], which limits their usage for the discovery of genetic variation. One genome, completed by a Pacific Biosciences (PacBio) sequencing strategy, produced 31 scaffolds [21]. PacBio long-read and Illumina short-read sequencing, combining with Hi-C analysis, has been used to assemble high-quality genomes for many plant species [22–24], but a chromosomal-level genome assembly of mushrooms is rare [25].

RAPD, AFLP and SSR, as well as other molecular markers, have been used for genetic diversity and population analysis. RAPD markers show that wild resources have abundant genetic diversity while many cultivated strains are genetically similar. This may result from the artificial selection that occurs during cultivation [26–29]. *L. edodes* population from mainland China, Taiwan and Japan clusters into distinct groups [30]. Most cultivated strains exhibit large genetic differentiation from wild strains [31]. Molecular markers have also been used to study the origination of *L. edodes* strains. Cultivated *L. edodes* strains in Korea, Japan and China originated in Northeast Asia [32]. The development of Next-Generation Sequencing (NGS) technologies permits cost-effective whole genome resequencing at a population scale, and have been used to map key genes related to important traits of plants and animals [33–35]. However, the technique is seldom used in mushrooms [36]. Therefore, high-throughput DNA sequencing is suitable for genetic research and the mapping of Cd accumulation genes in *L. edodes*.

*L. edodes* L808–1 is a strain that has been cultivated in China for than 30 years. In this study, we performed de novo genome assembly of *L. edodes* L808–1 using PacBio long-read and Illumina short-read sequencing, along with Hi-C technique. We conducted population genetics analysis based on genome-wide resequencing of 99 wild, domesticated and improved cultivars. The specific objectives included: (1) developing a high-quality reference genome for *L. edodes* cultivars that can be used for genome function analysis and genetic variation discovery, (2) determining the genetic architecture and breeding history of *L. edodes* germplasm collected from China and (3) identifying potential key genes related to Cd accumulation in *L. edodes*. The study could facilitate our understanding of the evolution and domestication of *L. edodes*.

## Results

### Chromosome-level genome construction for *L. edodes*

We assembled a high-quality 45.87 Mb genome for *L. edodes* using monokaryon strain L808–1, containing 46 major contigs (> 2 kb). The contig N50 and N90 sizes were 2.17 Mb and 0.8 Mb, respectively (Table 1). Over 96% of complete BUSCOs were detected in the genome, demonstrating the sequence continuity and completeness of the L808–1 genome. The alignment of next-generation sequencing (NGS) short reads resulted in a mapping ratio of 97.26%, indicating the high quality of the genome.

**Table 1:** Statistics for genome assembly. Note that numbers for N50 and N90 are sequence numbers accounting for the N50 and N90 statistics

	Scaffold		Contig	
	Length (bp)	Number	Length (bp)	Number
Maximum length	8,591,216	–	5,509,025	–
N50	4,935,280	4	2,167,243	8
N90	2,763,100	9	801,626	21
Total length	45,870,312	–	45,867,712	–
Number > =100 bp	–	25	–	51
Number > =2000 bp	–	21	–	46
GC rate	0.46		0.46	

**Fig. 1:**
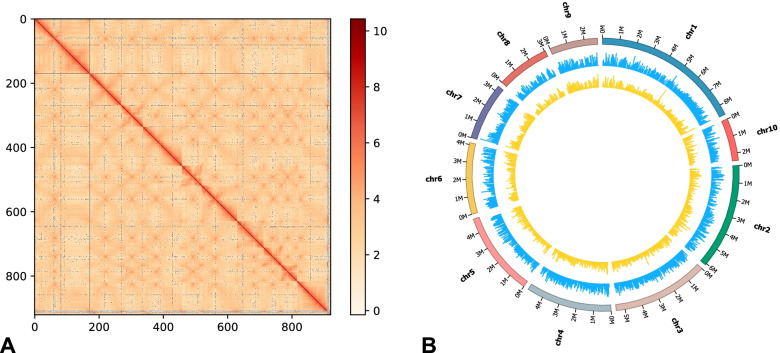
Hi-C interaction heat map and SNP distribution of the *L. edodes* genome. (**A**) Hi-C interaction heat map for *L. edodes*. The red and white schemes represent strong and weak interactions, respectively; (**B**) Circos plot for distribution and density SNPs (blue) and InDels (yellow) along the genome for *L. edodes* population. The lengths of chromosomes are shown by marks outside the circos

We used the Hi-C technique to construct the chromosome-level genome for *L. edodes* L808–1 (Fig. 1). We obtained 48.04 million paired reads with a total of 17.88 Gb for Hi-C sequencing. After contig sequence clustering, ordering and orientating, we successfully anchored 99.16% of the total genome sequences into 10 chromosomes. The scaffold N50 value was 4.94 Mb (Table 1), providing a high-quality chromosome-level genome for *L. edodes*.

We identified 27.89% (total of 12.84 Mb) of the L808–1 genome as repetitive elements using RepeatModeler and RepeatMasker (Table S1). All the major TE types were found, including DNA repeats, LINE, SINE and LTR. LTRs were the most abundant type representing 24.74% of the genome. DNA transposable elements comprised 0.74% of the genome, LINE accounted for 0.77% of the genome and 1.64% of the genome was unclassified repeats (Table S1).

For protein-coding gene annotation, homology-, de novo- and RNA-sequencing–based methods were used. We performed RNA sequencing on different developmental stages of *L. edodes* to obtain transcriptome data for gene prediction. Multiple tissue types, described above, were collected to cover as many tissue-specific transcripts as possible. After RNA-sequencing of the three key developmental stages (monokaryotic mycelia grown on substrates, mycelia form the brown film, mature fruiting bodies), low-quality reads and adapter sequences were removed from the raw data. The clean data were aligned to the *L. edodes* L808–1 genome. Then, we predicted 9853 genes with an average gene length of 3502 bp and an average of 7 exons (Table 2). To validate the gene annotation, we aligned RNA-Seq data to the reference genome with Hisat2 [37], which compared the mapping result to our gene models. Then, gene annotations in our genome were compared with RNA-Seq data using annotation edit distance (AED), where a higher AED represents a higher divergence of gene prediction and zero AED implies a perfect overlap of RNA-Seq and gene annotation [38]. Among all of the gene models, we found that 8864 predicted genes, accounting for 89.9% of the total genes in our annotation, were supported by RNA-Seq data, and more than 5000 genes had an AED index of 0 (Fig. S1). This indicated that these gene models totally overlapped with the RNA-Seq alignment.

**Table 2:** Comparison of *L. edodes* genomes with previous reported genomes. Note that accession is the sequence accession in NCBI

	L808–1	B17	W1–26	L54-A	L3	NBRC 111202	135A	135B
Accession	JABFYJ00 0000000	LSDU00 000000	LDAT00 000000	LOHM00 000000	MRUK00 000000	BDUG00 000000	ASZD00 000000	ASZE00 000000
Sequencing method	PacBio Sequel + Illumina + Hi-C	PacBio RSII + Illumina	Illumina HiSeq 2000	Roche 454	Illumina HiSeq	Illumina HiSeq 2500	Illumina	Illumina
Genome size (Mb)	45.87	46.11	41.82	38.54	40.93	39.92	36.69	37.64
number of scaffolds	10	31	340	2398	3043	1951	2997	3305
N50 (Mb)	4.94	3.66	0.30	0.03	0.06	0.05	0.05	0.05
GC content (%)	46.00	46.00	46.10	43.30	46.00	44.70	46.00	46.00
Number of genes	9853	13,426	14,889	–	–	12,050	–	–
Average gene length (bp)	2963	1612	2217	–	–	–	–	–
Reference	This work	[21]	[18]	[20]	Unpublished	[19]	Unpublished	Unpublished

Among all annotated genes, 9448 (95.89%) protein-coding genes were successfully functionally annotated, including 9421 (95.62%), 4919 (49.92%), 4177 (41.78%), 4155 (42.17%) and 4453 (45.19%), which could be annotated based on NCBI Nr databases, KEGG, Swiss-Prot, KOG and GO, respectively. For non-coding RNA species, 325 tRNAs, 35 rRNAs and 21 snRNAs were identified (Table S2).

### Genetic architecture analysis of 99 *L. edodes* strains

We used the L808–1 genome as the reference genome to conduct genomic sequencing reads alignments for the 99 *L. edodes* strains. The samples were collected primarily from China, Japan, the United States and Australia. A total of 34 wild strains were collected from China including northeast, central and southwest regions, and 65 cultivated strains were collected from other worldwide locations. A total of 120 Gb sequence data were generated from the 34 wild strains and 65 commercial cultivars. Genome alignment of *L. edodes* germplasm sequences indicated the sequencing depths were 22–40 X and the coverage was up to 99% relative to the assembled L808–1 genome. The mapping ratio of short-reads alignment ranged from 88 to 96% for samples (Table S3), indicating the monokaryon genome could be used to detect the majority of whole-genome markers for population studies. We found that wild strains generally exhibited lower mapping rates while cultivated strains were higher, illuminating the differentiation of wild and cultivated strains. After quality filtering and aligning the reads against the reference genome, we detected a total of 1,444,492 high-quality SNPs and 148,158 InDels that were generally evenly distributed along the whole genome (Fig. 1). We identified 153,998 nonsynonymous coding SNPs and 255,426 synonymous coding SNPs. These variants provide a new resource for *L. edodes* biology study and breeding.

**Fig. 2:**
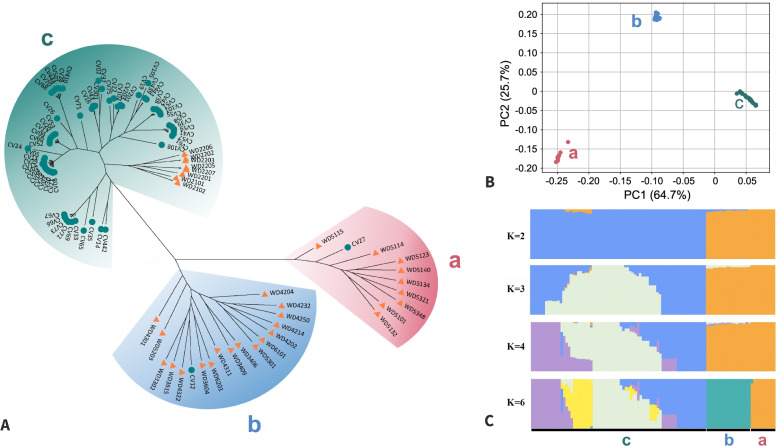
Analysis of genetic diversity of *L. edodes* population. (**A**) Phylogenetic tree using the whole-genome SNP information. All samples clustered into a, b and c groups. Wild and cultivated strains are labeled using yellow triangle and green dot, respectively; (**B**) the clustering of all samples using PCA analysis and (**C**) population genetic structure for all samples using genomic SNP loci

We performed genetic diversity and population structure analysis for 99 *L. edodes* strains. The neighbor-joining (NJ) tree revealed that the 99 resequenced *L. edodes* strains clustered into three groups. Group A included 9 wild strains from the plateau areas of Yunnan and Sichuan provinces in Southwest China and one cultivated strain (CV28) domesticated from a wild strain. Group B contained 16 wild strains from Central China and 1 cultivated strain (CV12) domesticated from a wild strain. Notably, we found 1 exception of a wild strain (WD5301) from Yunnan in group B, which should have clustered into Group A according to its original location. The reason for this result is unknown. Wild strains from Northeast China and most cultivated strains were clustered in Group C. Our phylogenetic analysis result was consistent with previous studies [39] showing that *L. edodes* strains in China are generally divided into three groups, namely, strains from northeast, central and southwest regions (Fig. 2). Principal component analysis (PCA) and population structure analysis provided additional corroborating evidence for these groupings. In the PCA analysis, the clustering of samples was consistent with neighbor-joining–based phylogenetic relationships. Notably, the top three components in the PCA analysis explained almost 100% of the variance among population (Fig. S2).

We also used ADMIXTURE [40] to infer the population structure of these samples. The population structures were consistent with phylogenetic and PCA analysis showing that the genetic relationships among wild samples were highly correlated with their geographical distribution. According to the result, K = 4 was a sensible modeling choice with the lowest cross-validation error (Fig. S3), indicating that the samples studied were likely formed via genetic admixture among four ancestral source population. Cultivated strains (Group C in Fig. 2A) exhibited a complex population structure compared to their closely related wild populations. This could be explained by artificial hybrid breeding among these strains.

### Breeding history for cultivated *L. edodes* strains in China

We selected 26 breeding varieties widely used in China with clearly recorded breeding histories. The breeding history diagram was constructed based on records for the cultivated strains and literature [41]. The 26 cultivated strains and their parental strains are shown in the breeding diagram, including 7402 (CV02), CR02 (CV16), 939 (CV72), shenxiang16 (CV33), L135 (CV65), senyuan10 (CV28), Senyuan8404 (CV27), Xiangza26 (CV13), N5 (CV41), HuxiangF2 (CV39), L808 (CV51) and Shengxiang215 (CV38).

Based on the whole genome sequence of *L. edodes* L808–1, 955,236 SNPs with high quality were screened out from 26 cultivated strains, with an average of 36,740 SNPs per individual. Among them, 225,928 (23.65%) SNPs were located in the intergenic spacer region, 109,741 (11.49%) in the intron region, 124,801 (13.06%) in the upstream region, 130,014 (13.61%) in the downstream region, 85,868 (8.99%) in the 3’UTR region, 86,276 (9.03%) in the 5’UTR region, and 836 (0.09%) in the shear donor. A total of 94,525 (9.90%) SNPs could cause non synonymous mutations and 131,823 (13.80%) SNPs could cause synonymous mutations (Table S4).

The phylogeny of the strains, based on whole-genome variants information, was consistent with the breeding history and could be used to validate the parental relationships for strains in breeding. For example, Senyuan8404 (CV27) was domesticated from wild strains so that CV27 was grouped together with wild population. Shengxiang215 (CV38) was selected from L808 (CV51), which is closely clustered in the phylogenetic analysis. Shenxiang16 (CV33) was selected by breeding of 939 (CV72) and L135 (CV65) so that the three strains were also grouped in the phylogenetic tree

**Fig. 3:**
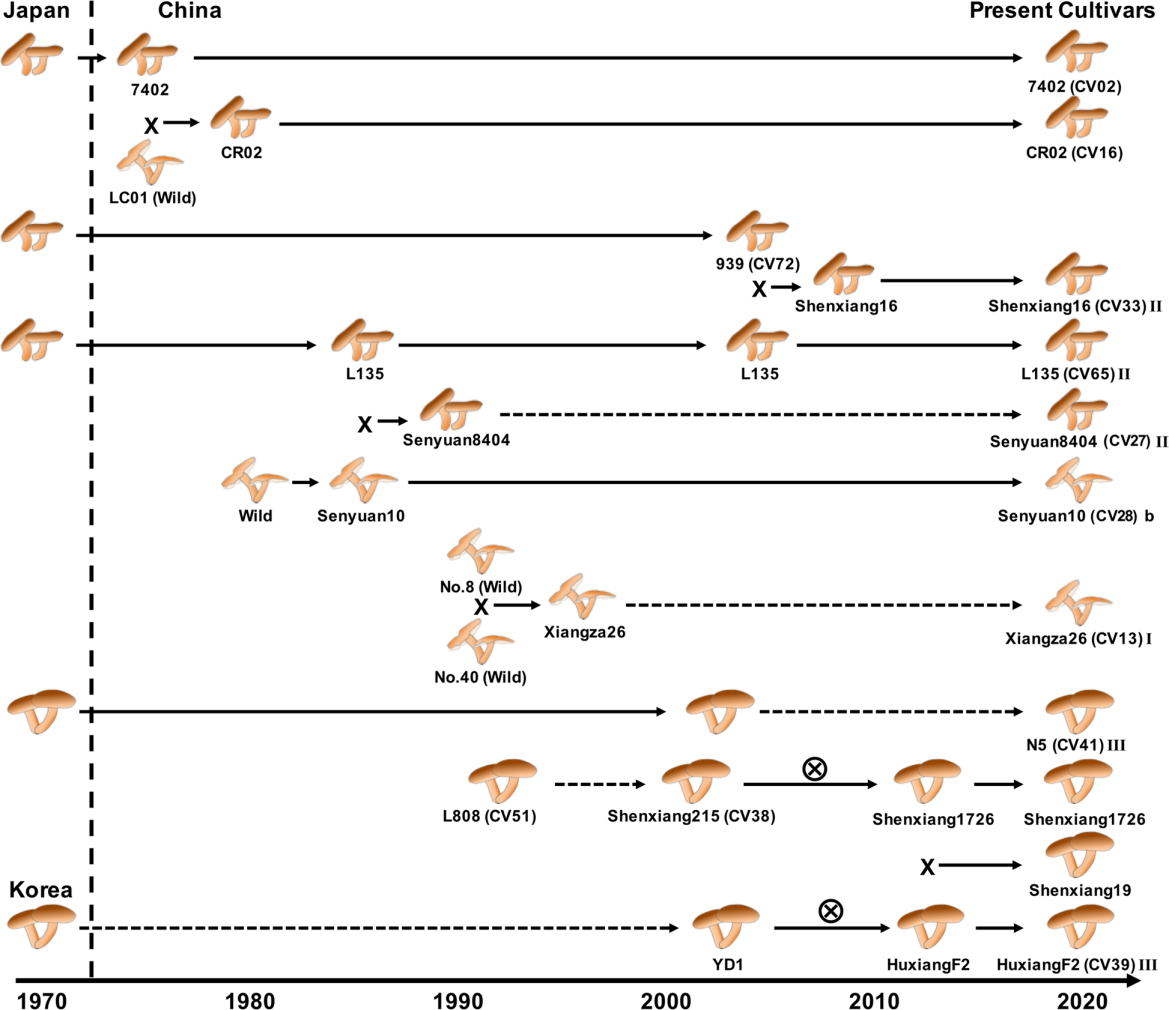
Genetic relationship of the main cultivated germplasm in China and their breeding history. Note that breeding history and relationships of strains were referenced from previous literature. Samples sequenced in this study are marked by their sample names in brackets. Group names in population analysis are shown after sample name, where b represented group b in the phylogenetic tree of Fig. 2, and I, II and III show subgroups in the population structure of 26 cultivars in Fig. S4

These 26 cultivated strains could be categorized into three groups according to their spawn running temperature and time. Group I mainly includes the high-temperature varieties of *L. edodes* in China, such as CV08 (CR04), CV11 (Wuxiang1), CV13 (Xiangza26). The growth temperature of the mycelium and fruiting body of Group I is higher than the other two groups, and there is a short period of spawn running. Group II mainly includes the long-term use of low-temperature and long period of spawn running time groups, including CV70(241–4), CV65(135) and CV67 (9015). Group III mainly includes cultivated strains with low temperature and median period of spawn running times, which are suitable for industry cultivation in controlled environments, such as CV39 (HXF2), CV58 (212) and CV19 (HX5). The three groups with distinct agricultural traits are genetically differentiated based on genetic structure analysis (Fig. S4), which may have resulted from their artificial selection. Clustering analysis from phylogenetic, PCA and population structure analysis (Fig. 2) showed that the majority of cultivated strains were closely related to the wild northeast population, suggesting that the cultivated strains in China might have originated from Northeast Asia. This is consistent with a previous study [32] and the fact that many strains, such as CV65 in Group II and CV41 and CV39 in Group III, were either Japanese varieties or their hybrids [41] (Fig. 3).

### Cd accumulation in *L. edodes*

**Fig. 4:**
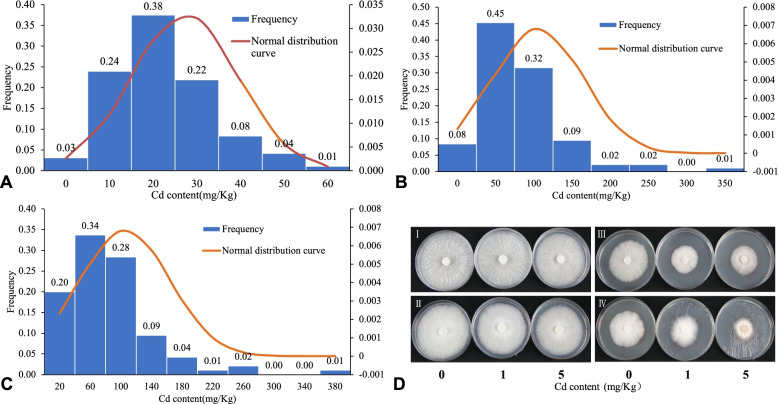
Frequency distribution of Cd content and phenotypic difference in *L. edodes* mycelium under Cd stress. (**A**)–(**C**) Frequency distribution of Cd content in mycelium under 0, 1 mg/kg and 5 mg/kg Cd stress, respectively. (**D**) Four main types of germplasm resources according to Cd content and affects on mycelial growth under Cd stress. I: Low Cd enrichment and slightly affected; II: high Cd enrichment but only slight effects on growth; III: low Cd enrichment but severely affected; IV: high Cd enrichment and severely affected

The Cd contents of 99 *L. edodes* strains exposed to Cd at 1 mg/kg and 5 mg/kg in the PDA medium were determined (Table S5). Significant differences of Cd content in the mycelium of different germplasm occurred under Cd stress, and the Cd content increased with increased Cd concentration. The distribution of Cd content in mycelium in the 1 mg/kg and 5 mg/kg treatments was consistent with the normal distribution (Fig. 4). When the concentration was 1 mg/kg, Cd was detected in the majority of strains (0–70 mg/kg), among which the highest Cd content was WD6201 (66.39 mg/kg) and the lowest was WD3406 (0.448 mg/kg). The Cd concentration for several strains, such as WD3615, was not determined, because the experiment did not provide sufficient mycelia for Cd measurement. When the concentration was 5 mg/kg, the highest value was CV59 (383.5 mg/kg) and the lowest was CV60 (27.33 mg/kg). The results indicated substantial variation among the 99 *L. edodes* strains in the amount of Cd absorbed.

### Genome-wide association study (GWAS) of cd accumulation of *L. edodes*

**Fig. 5:**
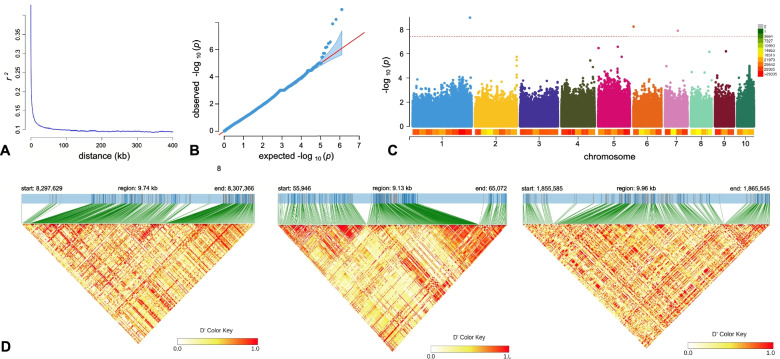
GWAS analysis results with Cd stress concentration of 1 mg/kg. (**A**) Linkage disequilibrium (LD) decay for SNP loci over distance for association population along the *L. edodes* genome; (**B**) QQplot of GWAS for 1 mg/kg Cd Stress. X- and Y-axis represented expected and observed p-value, respectively; (**C**) Manhattan plots of association result for GWAS analysis for 1 mg/kg Cd Stress. The Y-axis is the negative of logarithm of *p*-value for GWAS for SNP loci along chromosomes (X-axis). The dashed line represents the significant threshold for the GWAS analysis from the Bonferroni correction method. (**D**) LD blocks of Cd stress-related SNPs in the three chromosomes. Strong and weak linkages are shown using red and white color schemes, respectively

Using the SNP data with a minor allele frequency (MAF) ≥0.05 of 99 samples of *L. edodes* germplasm, GWAS analysis of the Cd accumulation trait was conducted using FarmCPU of GAPIT. Three SNPs were significantly associated with Cd accumulation traits when the Cd concentration in the mycelium reached 1 mg/kg, while there was no significantly correlated locus when mycelium levels reached 5 mg/kg. Manhattan plots showed three associated SNPs on chromosomes 1, 6 and 7, respectively. All three SNPs occurred in three different gene coding regions. LD decay analysis results showed that the LD decay rate of *L. edodes* population was extremely fast, making the LD region near the three associated SNPs contain fewer genes that would reduce the complexity of candidate gene screening. Therefore, we performed LD block analysis within 5 K/10 K from the three associated SNP region and found four additional candidate genes. According to the gene functional annotations, two of the seven genes were annotated as DNA ligase (MAKER_DHR6000170) and aminoacyl tRNA synthetase (MAKER_DHR7000053), while the other five genes were not functionally annotated (Fig. 5).

## Discussion

A highly continuous, complete and accurate reference genome provides a basis for exploring species evolution, genetic variation and functional gene mapping. The multi-level strategy combining “PacBio + Illumina + Hi-C” was successfully applied to chromosome genome assembly of pepper [42], barley [43], yellow croaker [44], goat [45], puffer [46] and other animals and plants but has rarely been used for macrofungal species [25]. We used the strategy on the fungus of *L. edodes* L808 to obtain a high-quality reference genome at the chromosome level. Compared to seven genomes of *L. edodes* in the NCBI genome database (Table 2), the assembled L808–1 reference genome showed high continuity and integrity. The majority (99%) of the genome sequences of L808–1 were successfully anchored upon 10 chromosomes. We validated the improved continuity of the L808–1 genome, where more than 90.32% contigs of the B17 strain [21] were reliably mapped to L808–1 with alignment ratios greater than 72.51% (Fig. S5). Although the B17 strain was also assembled by PacBio long reads, our assembly resulted in a contig N50 length of 2.17 Mb with 46 major contigs, while the contig N50 length was 858 kb with 74 contigs for the B17 strain. We also applied the Hi-C technique to scaffold the genome to chromosomal level (10 scaffolds), but the genome for the B17 strain only used sequencing reads to link contigs to 31 scaffolds. We predicted 9853 genes in the L808–1 genome, which was fewer than previously studies. Although the B17 strain predicted 13,028 gene models, only 10,700 genes could hit homologs in the public databases. Recent genome studies on Phellinus gilvus, Pleurotus ostreatus and Hericium erinaceus using long-read assembly resulted in 9982, 11,875 and 10,620 genes, respectively, which is comparable with the 9853 predicted genes in our study. Therefore, we obtained a high-quality reference genome for *L. edodes,* which lays a solid foundation for analyzing the genetic architecture and functional gene mapping of *L. edodes*.

Based on genome-wide resequencing of 60 *L. edodes* strains, Xiao et al. [31] showed that the *L. edodes* germplasm in China has different genetic backgrounds. We included wild strains of *L. edodes* from different ecological regions in China and cultivated varieties with short spawn periods in Northeast China. Based on genotyping information from whole genome resequencing data, the phylogenetic analysis showed that all samples were clustered into three groups and the PCA analysis showed that PC1 and PC2 accounted for 64.7 and 25.7% of the total variances for these samples, respectively. The three groups included wild strains from Yunnan and Sichuan Plateau areas in Southwest China, wild strains from central China, wild strains from Northeast China and most of the cultivated strains. The genetic analysis results showed that wild strains exhibited high genetic diversity, while less gene flow occurs among wild strains from different ecological regions of China. We also found unique allele and genetic backgrounds in wild strains distributed in Southwest and Northeast China. Therefore, these wild strains in China contain unique gene pools that could be used as breeding resources for strain improvement of commercial *L. edodes*.

*L. edodes* has been cultivated in China for at least 800 years but historical breeding records were not revealed until now. The genetic background of many common varieties is unclear, and their breeding history is poorly understood. According to the breeding history of *L. edodes* varieties in China and population analysis based on genome-wide resequencing, we suggest that there are two main sources of the Chinese varieties of *L.* edodes. First, strains might have been directly domesticated from wild strains collected from different ecological regions of China, such as CV27 and CV12. Second, strains might also have originated from the Northeast Asian regions. These include strains such as CV41, CV39 and CV65 that were introduced or crossbred from Japanese varieties. We also found that the cultivated varieties in China exhibited genetic differentiation, which was consistent with their distinct differences in spawn running temperature and time. The whole-genome data for those strains provide resources that could facilitate the identification of functional genes for spawn running temperature and time and the efficient usage of cultivated and wild germplasm in *L. edodes* breeding.

The molecular mechanism of Cd accumulation in edible mushrooms is unclear. GWAS has been used to find genes related to agronomic traits in animals and plants at the whole genome level [47–50]. However, there are few reports on its application in mushrooms. To control false positives, it is important to select a powerful and efficient model for GWAS analysis. In this study, Fixed and random model Circulating Probability Unification (FarmCPU) was used for GWAS analysis. Compared to the traditional mixed linear model (MLM), the FarmCPU model improves statistical power and reduces computing time. This is achieved by iterative usage of the fixed effects and random effects models [51]. In this study, we first performed GWAS analysis of Cd accumulation of different *L. edodes* strains using MLM, but no significant associated loci were found under the Cd stress concentrations of 1 mg/kg and 5 mg/kg. However, we found three significant associated loci under 1 mg/kg Cd stress using FarmCPU. We also used the FarmCPU to find six significantly associated loci involving eight genes related to the mycelia growth in the sawdust culture medium of different *L. edodes* strains (unpublished). Therefore, we believe that the FarmCPU model could be used for GWAS analysis to mine candidate genes related to agronomic and economic traits of mushrooms.

Our GWAS analysis showed that genes encoding DNA ligase and aminoacyl tRNA synthetase were candidate genes related to Cd accumulation in *L. edodes*. DNA ligase is an important enzyme for DNA damage repair [52], indicating that Cd accumulation might be related to the DNA damage repair system in *L. edodes*. Aminoacyl tRNA synthetase has been reported in many studies involving Cd stress in plants. Hu et al. [53] demonstrated that the metabolites increased in aminoacyl tRNA biosynthesis and cyanoamino acid metabolism under Cd stress of rice. DNA damage is a common result of Cd toxicity to organisms. Studies in *Arabidopsis thaliana* and other model plants showed that Cd toxicity causes DNA single strand damage through the production of reactive oxygen species and inhibiting the scavenging effect of superoxide dismutase and other enzymes [54]. Yang et al. [55] found that lead stress led to the imbalance of active oxygen metabolism in amaranth, which aggravated peroxidation and hindered amaranth growth. This result was consistent with a previous study [36] showing that the activities of antioxidant enzymes were associated with Cd accumulation for *L. edodes*. Therefore, our current results lay a foundation for analyzing the molecular mechanism of Cd accumulation of *L. edodes* and other mushrooms.

## Conclusions

We accomplished a high-quality genome assembly of *L. edodes* with high-depth sequencing data. Population genetics analysis for 99 wild and cultivated *L. edodes* was performed. The long-term breeding history of *L. edodes* in China was analyzed using whole-genome information. Using GWAS analysis, three loci and two genes were identified that might be involved in the Cd accumulation of *L. edodes*. This study provides a valuable resource for future genetics and breeding studies for *L. edodes.*

## Materials and methods

### Sample collections for de novo genome assembly of *L. edodes*

*L. edodes* strain L808 was selected for de novo genome sequencing and maintained in the Improved and Standardized Spawn Breeding Center (ISSBC), Shanghai Academy of Agricultural Sciences, China. One monokaryon (L808–1) of the dikaryon strain L808 was originally isolated by protoplast monokaryotization [56]. The obtained monokaryon L808–1 was cultured on 20 Potato Dextrose Agar (PDA) plates covered with cellophane at 25 °C in darkness for 14 days. These mycelia were then collected, frozen in liquid nitrogen and used for genome sequencing and chromosome-level genome construction.

### DNA extraction and sequencing for de novo genome assembly

Genomic DNA sequencing for de novo assembly was performed using Illumina and PacBio (Pacific Biosciences, CA, USA) sequencing. Genomic DNA was extracted from mycelia of the monokaryon strain L808–1 using a DNA extraction kit (CWBiotech Corporation, Beijing, China) according to manufacturer instructions. The genomic DNA was examined by agarose gel electrophoresis to analyze the purity and integrity, and detected with a Qubit fluorometer (Invitrogen, CA, USA). The total DNA quantity used for PacBio sequencing was > 10 μg, and the DNA concentration was > 80 ng/μL. The value of OD 260/280 was ranged between 1.7 and 2.0 and OD 260/230 was between 1.8 and 2.2. After the test was qualified, the Illumina HiSeq X ten platform (Illumina, CA, USA) was used to generate short genomic reads using construction of the paired-end library with an insert size of 250 bp following manufacturer’s protocol. We obtained 4 Gb of short genomic reads with ~90X coverage of the genome (Table 3). The High-Throughput Quality Control (HTQC) utility was used to filter the adapter sequences and low quality reads [57]. The cleaned reads were used for the subsequent analysis. For PacBio sequencing, the genomic DNA was segmented and size-selected for 20 kb using BluePippin (Sage Science, Inc., Beverly, MA, USA). After the end repair, we connected the joint at both ends of the clip to prepare the DNA library. To improve library quality, we removed single-stranded ends from fragments using ExoVII Treatment of the DNA step. The library was sequenced on the PacBio Sequle platform for 10 h. All of the genomic sequencing data used for genome assembly are summarized in Table 3. The high-throughput chromosome conformation capture technique (Hi-C) was then used to construct the chromosome-level genome assembly for *L. edodes* L808–1 (Fig. 1). The cultured mycelia were used to construct the Hi-C library and sequenced according to previously reported methods [58, 59].

**Table 3:** Genome and transcriptome sequencing data for *Lentinula edodes* strain L808. Note that coverage is calculate using final assembled genome size

Source	Samples	Platform	Data (Gb)	Coverage (x)	Application
Genome	1	Illumina X Ten	~ 4.0	86.9	Genome polish
Genome	1	PacBio Sequel	~ 3.5	76.9	Genome assembly
Transcriptome	12	Illumina X Ten	~ 6.2*12	–	Genome annotation
Hi-C	1	Illumina X Ten	~ 17.9	388.9	Chromosome assembly
Genome	99	Illumina X Ten	~ 1.1*99	24.7	Population genetics

### Transcriptome sequencing

Three major developmental stages (monokaryotic mycelia grown on substrates, mycelia form the brown film and mature fruiting bodies) of the *L. edodes* strain L808 were selected for transcriptome sequencing. Two protoplast monokaryons (L808–1 and L808–2) of the dikaryon strain L808 were cultured on PDA plates covered with cellophane at 25 °C in darkness. After 10 days, the mycelia were fully grown and colonized in each plate. We randomly collected the mycelia from nine plates and froze the mycelia in liquid nitrogen (Stage I). Then we cultivated the *L. edodes* strain L808 in the Shanghai Yinghui mushroom factory. The cultivation substrates contained 1400 g of medium composed of 79% sawdust, 20% bran and 1% gypsum. All of the RNA-Seq samples were selected from one factory batch. We initially cultured L808 in PDA solid medium at 25 °C for 10 days, and then transferred this to cultivation substrates in polyethylene bags with a microfilter breathing window. The bags were maintained for 45 days in darkness and another 45 days under light at 25 °C. Then the cultivation bags were stimulated at 15 °C for 24 h to promote fruiting. After stimulation, the bags were cultivated at 22 °C under light for primordial initiation. We randomly sampled the small primordia into the corresponding nine tubes (Stage II). After five d, the primordia differentiated into stipes and caps. Then the fruiting bodies were shaped, and we selected nine mature fruiting bodies (Stage III). All of the collected samples of the three developmental stages were stored at − 80 °C prior to RNA extraction. Total RNA from four samples for each development stage, resulting in a total 12 samples. The samples were separately extracted by TRIzol reagent (Invitrogen, CA, USA), including mycelium and fruiting body tissues. RNA quality was tested with a 2100 Bioanalyzer (Agilent Technologies, CA, USA) and NanoDrop ND-1000 (Labtech, Ringmer, UK). A total of 12 cDNA libraries were constructed and conducted on the Illumina HiSeq X Ten platform using paired-end sequencing. All the RNA sequencing data are summarized in Table 3.

### De novo genome assembly of *L. edodes* and construction of the chromosome-level genome

The procedures of de novo genome assembly of *L. edodes* L808–1 included self-correction of long reads, correction of read alignment, construction of the sequence graph and contig assembly. PacBio long reads were assembled by SMARTdenovo (https://github.com/ruanjue/smartdenovo) using the default parameters. The final genome sequences were corrected by one round of polishing using long sequencing reads by Arrow [46] and two rounds of polishing using NGS short reads by Pilon [60]. BUSCO and short-read mapping pipelines were used for quantitative assessment of the genome assembly of *L. edodes*.

We used Bowtie 1.2.2 [61] to map the Hi-C sequencing reads to the polished *L. edodes* L808–1 genome. The two read ends were independently aligned to the L808–1 genome, and the uniquely aligned ends of the read pairs were then selected. We further combined the previously reported methods and hiclib Python library [62] to filter the Hi-C reads and quantified and normalized the interaction frequencies among contigs. Using the interaction matrix among sequences, we then clustered contigs with the agglomerative hierarchical clustering method of Lachesis [63] using default parameters.

### Gene model prediction and functional annotations

To search transposon sequences, the assembled genome of *L. edodes* L808–1 was aligned to the *transposon* Repbase database with RepeatMasker (version 3.3.0) and RepeatProteinMasker software [64]. Tandem Repeat Finder [65] was used to predict the tandem repeat sequences (Table S1). The rRNA sequences were detected by both sequence homology and de novo prediction with rRNAmmer software [66]. The tRNA genes were detected using tRNAscan-SE [67]. Non-coding RNA genes, such as snRNA and miRNA, were predicted by Rfam software [68].

De novo gene prediction using AUGUSTUS v2.5.5 [69] and GlimmerHMM 3.0.1 [70] was trained using the model species *Coprinopsis cinerea* and created training sets. Then, protein sequences of *Agaricus bisporus*, *Coprinopsis cinerea*, *Pleurotus ostreatus* and *Schizophyllum commune* were mapped to the *L. edodes* L808–1 genome by TBLASTN and analysed with exonerate [71]. In addition, the time-series transcriptome sequences obtained from the IIlumina sequencing platform were mapped to the L808–1 genome by Hisat2 [37], and gene structure was predicted by Cufflinks [72]. We merged the three gene models and removed redundancy by MAKER [73]. The result predicted 9853 protein-coding genes (Table S6).

Putative gene functions were compared with the non-redundant (NR) database of NCBI, Swiss-Prot databases, and Eukaryotic Clusters of Orthologous Groups (KOG) by BLAST (e < 1.00E^− 5^). Gene motif and domains were determined with InterProScan [74]. Gene Ontology (GO) annotation was obtained using Blast2GO 2.5.0 [75]. Kyoto Encyclopedia of Genes and Genomes (KEGG) (http:/www.kegg.jp/) annotation was conducted by the BLASTX algorithm of DIAMOND program (e < 1.00E^− 5^) (Table S2).

### Whole genome resequencing and population genetic analysis

We conducted whole genome resequencing of total 99 strains using NGS technology on the Illumina HiSeq X Ten platform. A total of 34 wild strains were collected from China including northeast, central and southwest regions, and 65 cultivated strains were collected from worldwide locations including China, Japan, the United States and Australia. For the wild strains, 8 strains were collected from northeast China including Liaoning and Jilin province; 17 strains were collected from central China, including Anhui, Zhejiang, Jiangxi, Hubei, Hunan, Shaanxi, Gansu and Guizhou provinces; and 10 strains collected from southwest China including Sichuan and Yunnan provinces. We cultured all strains on five MYG (malt extract 0.5%, yeast extract 0.25%, glucose 1.0% and agar 2.0%) solid medium covered cellophane sheets for 10 d.

We used FastQC (http://www.bioinformatics.babraham.ac.uk/projects/fastqc/) and HTQC (https://sourceforge.net/projects/htqc) to clean the raw reads generated from NGS technology [57]. We first filtered the adapter sequence and then filtered reads with more than 10% N bases. We filtered any ends with more than 50% inferior quality (≤5) bases. Burrows-Wheeler Aligner (BWA) software was used to align the high-quality reads to the *L. edodes* L808–1 genome [76]. The SNPs (single-nucleotide polymorphisms) and short insertions and deletions (InDels, length < 100 bp) were identified by GATK4.0. The obtained high-quality SNPs were also used in the SNP summary and regions of homozygosity analyses. The SNP calls were calculated by Analysis of Next Generation Sequencing Data (ANGSD v.0.902) [77].

Principal component analysis (PCA) for the 99 strains was conducted by smartpca in EIGENSOFT v5.0 [78] (Fig. 2). Using the outgroup *Coprinopsis cinerea*, a neighbor-joining (NJ) tree was constructed for the 99 strains using TreeBeST v1.9.2 software (http://treesoft.sourceforge.net/treebest.shtml) with 200 fast bootstrap replicates. To investigate population admixtures, the number of genetic clusters inherent in the genomes of population was investigated using K from 2 to 6 with ADMIXTURE v1.3.0 [40].

### Evaluation of the cd accumulation of *L. edodes* germplasm

The 99 strains were inoculated on the PDA medium covered with cellophane at a Cd concentration of 1 mg/kg and 5 mg/kg and cultured for 20 days. Then, three biological repeats were collected to analyze the Cd content in the mycelium of the *L. edodes* germplasm according to the Chinese National Standards GB5009.15–2014. Sample pretreatment was conducted by microwave digestion [79]. Briefly, 0.5 g sample powder was weighed on an analytical balance, put into a microwave digestion tank, and 5 mL of concentrated nitric acid and 2 mL hydrogen peroxide were added. After digestion overnight, the digestion tank was cooled and opened. The digestive solution was colorless or light yellow, and the acid was heated to near dry. The digestion tank was rinsed three times with a small amount of 1% nitric acid solution, and the solution was transferred to a 25 mL volumetric bottle. The volume was fixed to the scale with 1% nitric acid solution, the mixture was mixed well and prepared for use. At the same time, a reagent blank test was conducted. The atomic absorption spectrophotometer AZ3000 (HITACHI, Tokyo, Japan) was used for determination.

### Genome-wide association analysis

Plink 1.90 beta [80] was used to conduct the initial quality control (parameters: geno = 0.1, mind = 0.1, maf = 0.05) and markers on chromosomes 1–10 were retained. Genotype imputation was performed by Beagle v4.0 [81], followed by a second quality control (Plink 1.90 beta, parameter: maf = 0.05). Finally, 99 strains and 1,273,019 markers were retained for GWAS. Association analysis was conducted with two models, Fixed and random model Circulating Probability Unification (FarmCPU) and Mixed Linear Model (MLM), with each containing the top three principal components (PCs) as covariates. LD decay analysis was performed using PopLDdecay [82]. LDblockShow [83] was used to perform LD block analysis within the 5 K/10 K nearby region. The physical location of significantly associated SNP and its corresponding adjacent SNP loci were compared with the L808–1 genome. The associated gene functions were annotated using the annotation dataset of the L808–1 genome. Manhattan map and QQ-plot were used to display the results graphically.

## Supplementary Information

**Additional file 1.**

**Additional file 2.**

## Data Availability

The sequencing dataset and genome assembly were deposited in public repositories. The genomic Illumina sequencing data, genomic PacBio sequencing data and genomic Hi-C sequencing data for strain L808–1, as well as the time-series transcriptome and whole-genome sequencing for *L. edodes* data, were deposited under accession number of SRP256131(https://www.ncbi.nlm.nih.gov/sra/?term=SRP256131). The final chromosome assembly and genome annotation were submitted to the NCBI Assembly Database, with accession number JABFYJ000000000(https://www.ncbi.nlm.nih.gov/assembly/GCA_015476405.1).
