# Supplementary material for: Chromosomal genome and population genetic analyses to reveal genetic architecture, breeding history and genes related to cadmium accumulation in Lentinula edodes
Source: BMC Genomics. 2022 Feb 10;23:120. doi: 10.1186/s12864-022-08325-x (PMC8832684; doi:10.1186/s12864-022-08325-x)
Supplement: Supplementary file 1 — Additional file 1. [file 12864_2022_8325_MOESM1_ESM.docx]

**Table S1. Repetitive elements annotation in the *Lentinula edodes* strain L808 genome.**

|  | **Repbase TEs** | | **TE proteins** | | ***De novo*** | | **Combined TEs** | |
| --- | --- | --- | --- | --- | --- | --- | --- | --- |
| Type | Length (bp) | % in genome | Length (bp) | % in genome | Length (bp) | % in genome | Length (bp) | % in genome |
| DNA | 61,236 | 0.13 | 13,140 | 0.03 | 278,097 | 0.6 | 342,188 | 0.74 |
| LINE | 68,296 | 0.15 | 91,390 | 0.2 | 292,014 | 0.63 | 353,341 | 0.77 |
| SINE | 591 | 0 | 0 | 0 | 56,708 | 0.12 | 57,299 | 0.12 |
| LTR | 2,719,864 | 5.91 | 3,646,982 | 7.92 | 11,069,521 | 24.05 | 11,385,579 | 24.74 |
| Satellite | 4,264 | 0.01 | 0 | 0 | 13,568 | 0.03 | 17,832 | 0.04 |
| Simple repeat | 5,199 | 0.01 | 0 | 0 | 0 | 0 | 5,199 | 0.01 |
| Other | 55 | 0 | 0 | 0 | 0 | 0 | 55 | 0 |
| Unknown | 10,618 | 0.02 | 0 | 0 | 744,223 | 1.62 | 754,805 | 1.64 |
| Total | 2,865,083 | 6.23 | 3,751,377 | 8.15 | 12,393,304 | 26.93 | 12,836,022 | 27.89 |

**Table S2. Functional annotation for protein-coding genes in the L808-1 genome.**

| **Type** | **Database** | **Number** | **Percentage (%)** |
| --- | --- | --- | --- |
| Total |  | 9,853 | 100 |
| Annotated genes | NR | 9,421 | 95.62 |
|  | Swiss-Prot | 4,117 | 41.78 |
|  | KOG | 4,155 | 42.17 |
|  | GO | 4,453 | 45.19 |
|  | KEGG | 4,919 | 49.92 |
| Unannotated genes |  | 405 | 4.11 |

**Table S3. Mapping statistics for strains. MapRate, MapPERate, Insert, Coverage, DeepMean represents mapping rate, paired mapping rate, insert length, mapping coverage and mean mapping depth, respectively.**

| Sample | PEreads | MapRate | MapPERate | Insert | Coverage | DeepMean |
| --- | --- | --- | --- | --- | --- | --- |
| CV02 | 8,952,806 | 92.87% | 92.29% | 285.6 | 99.92% | 28.3 |
| CV03 | 11,034,388 | 93.56% | 93.05% | 298.5 | 99.95% | 34.47 |
| CV04 | 10,411,588 | 93.73% | 93.17% | 299.3 | 99.95% | 32.57 |
| CV05 | 9,444,614 | 93.90% | 93.34% | 297.1 | 99.95% | 29.65 |
| CV06 | 9,147,804 | 92.33% | 91.78% | 285.7 | 99.95% | 28.26 |
| CV07 | 9,421,126 | 93.29% | 92.75% | 297.2 | 99.95% | 29.36 |
| CV08 | 9,036,320 | 94.58% | 94.05% | 297.6 | 99.95% | 28.47 |
| CV09 | 9,146,996 | 93.69% | 93.14% | 304.2 | 99.95% | 28.54 |
| CV101 | 12,556,806 | 95.26% | 94.84% | 297.7 | 100.00% | 38.51 |
| CV102 | 9,554,832 | 95.26% | 94.85% | 312.1 | 99.98% | 29.78 |
| CV105 | 8,438,478 | 94.10% | 93.56% | 302.6 | 99.95% | 26.49 |
| CV108 | 9,221,810 | 94.41% | 93.94% | 292.8 | 99.95% | 28.97 |
| CV11 | 8,641,382 | 91.66% | 91.08% | 210.2 | 99.95% | 25.63 |
| CV12 | 9,270,654 | 92.27% | 91.42% | 305.2 | 99.83% | 29.6 |
| CV13 | 10,256,762 | 92.71% | 92.17% | 275.6 | 99.95% | 31.65 |
| CV14 | 11,066,648 | 92.38% | 91.76% | 278.5 | 99.93% | 34.15 |
| CV16 | 10,972,396 | 93.72% | 93.24% | 298.2 | 99.96% | 34.38 |
| CV17 | 9,431,352 | 92.79% | 92.22% | 304.3 | 99.95% | 29.13 |
| CV19 | 10,742,078 | 93.77% | 93.27% | 293.1 | 99.94% | 33.73 |
| CV20 | 8,201,000 | 92.89% | 92.30% | 290.3 | 99.95% | 25.42 |
| CV201 | 12,945,716 | 95.83% | 95.45% | 275.4 | 99.98% | 40.34 |
| CV203 | 9,676,408 | 96.06% | 95.72% | 259.5 | 99.98% | 30.32 |
| CV21 | 8,227,920 | 93.37% | 92.85% | 286.6 | 99.95% | 25.71 |
| CV22 | 10,068,528 | 95.67% | 95.34% | 302.3 | 100.00% | 31.08 |
| CV24 | 9,498,706 | 93.47% | 92.94% | 302.9 | 99.96% | 29.56 |
| CV26 | 9,837,614 | 93.51% | 92.84% | 314.3 | 99.94% | 30.69 |
| CV27 | 10,931,292 | 92.91% | 92.21% | 304.2 | 99.92% | 34.25 |
| CV28 | 9,772,418 | 90.94% | 89.84% | 391.3 | 99.75% | 30.55 |
| CV29 | 8,164,930 | 92.69% | 92.12% | 288.2 | 99.95% | 25.31 |
| CV30 | 10,579,900 | 93.29% | 92.74% | 291.2 | 99.95% | 32.93 |
| CV301 | 10,360,270 | 93.89% | 93.41% | 301.8 | 99.96% | 32.51 |
| CV32 | 9,138,046 | 94.06% | 93.52% | 304.2 | 99.96% | 28.57 |
| CV33 | 10,423,876 | 93.05% | 92.42% | 305.8 | 99.92% | 33.06 |
| CV35 | 10,273,054 | 94.22% | 93.76% | 310.7 | 99.96% | 32.13 |
| CV38 | 8,206,236 | 95.37% | 94.97% | 306.8 | 100.00% | 25.22 |
| CV39 | 8,720,204 | 93.71% | 93.20% | 301.5 | 99.96% | 27.11 |
| CV41 | 10,836,492 | 95.14% | 94.67% | 302.5 | 99.96% | 34.18 |
| CV410 | 8,588,902 | 92.82% | 92.16% | 304.6 | 99.92% | 27.03 |
| CV42 | 9,650,602 | 95.03% | 94.63% | 298.6 | 100.00% | 29.54 |
| CV43 | 10,654,772 | 94.01% | 93.50% | 290.3 | 99.96% | 33.38 |
| CV439 | 10,268,146 | 94.58% | 94.17% | 263 | 99.96% | 31.95 |
| CV442 | 9,722,858 | 94.54% | 94.05% | 262.8 | 99.92% | 31.15 |
| CV443 | 8,867,338 | 95.26% | 94.80% | 276.5 | 99.96% | 27.84 |
| CV444 | 10,637,914 | 93.33% | 92.74% | 270.7 | 99.92% | 33.43 |
| CV501 | 10,943,488 | 92.82% | 92.24% | 264.4 | 99.92% | 34.12 |
| CV51 | 9,941,726 | 94.74% | 94.33% | 305.3 | 100.00% | 30.34 |
| CV52 | 10,366,522 | 92.68% | 92.11% | 304.1 | 99.96% | 32.01 |
| CV53 | 8,997,634 | 93.23% | 92.71% | 273.9 | 99.95% | 27.94 |
| CV54 | 9,039,938 | 93.19% | 92.65% | 292.4 | 99.95% | 28.14 |
| CV55 | 11,186,280 | 95.46% | 95.05% | 306.2 | 100.00% | 34.38 |
| CV57 | 8,929,172 | 94.54% | 94.06% | 283.6 | 99.96% | 28.02 |
| CV58 | 8,413,272 | 94.84% | 94.38% | 288.4 | 99.96% | 26.49 |
| CV59 | 10,611,514 | 93.50% | 93.01% | 305 | 99.96% | 32.84 |
| CV60 | 11,359,778 | 94.25% | 93.73% | 303.6 | 99.96% | 35.61 |
| CV61 | 9,390,488 | 94.77% | 94.28% | 291.2 | 99.96% | 29.52 |
| CV65 | 9,558,516 | 93.90% | 93.34% | 279.4 | 99.92% | 30.4 |
| CV66 | 10,651,290 | 92.97% | 92.36% | 296.6 | 99.92% | 33.71 |
| CV67 | 10,931,026 | 93.17% | 92.55% | 299.5 | 99.92% | 34.69 |
| CV68 | 8,894,116 | 92.16% | 91.43% | 303.1 | 99.92% | 27.8 |
| CV69 | 10,839,520 | 93.38% | 92.74% | 294.3 | 99.92% | 34.48 |
| CV70 | 10,728,844 | 92.62% | 91.94% | 301.8 | 99.92% | 33.56 |
| CV71 | 10,130,928 | 93.49% | 92.95% | 279.5 | 99.95% | 31.5 |
| CV72 | 10,770,670 | 92.76% | 92.20% | 291.6 | 99.92% | 34.02 |
| CV73 | 10,788,138 | 90.10% | 89.48% | 302.8 | 99.92% | 33.07 |
| CV75 | 7,976,774 | 93.01% | 92.50% | 299.6 | 99.96% | 24.87 |
| WD2101 | 10,449,404 | 92.72% | 92.12% | 293.7 | 99.92% | 32.94 |
| WD2102 | 10,662,630 | 88.83% | 88.23% | 228.4 | 99.93% | 30.99 |
| WD2201 | 10,657,148 | 88.08% | 87.51% | 300.3 | 99.92% | 32.04 |
| WD2202 | 10,242,992 | 94.91% | 94.36% | 295.9 | 99.92% | 33.15 |
| WD2203 | 8,880,888 | 94.96% | 94.42% | 291.5 | 99.92% | 28.84 |
| WD2205 | 11,502,690 | 92.64% | 91.80% | 307 | 99.92% | 35.49 |
| WD2206 | 12,369,862 | 92.91% | 92.26% | 301.3 | 99.92% | 38.8 |
| WD2207 | 8,574,224 | 92.78% | 91.98% | 296.7 | 99.92% | 26.6 |
| WD3302 | 8,697,140 | 89.79% | 89.01% | 281.6 | 99.84% | 26.97 |
| WD3406 | 9,778,656 | 92.34% | 91.53% | 285.9 | 99.83% | 31.17 |
| WD3409 | 9,308,752 | 92.33% | 91.67% | 283.2 | 99.84% | 29.56 |
| WD3604 | 10,624,634 | 91.76% | 90.85% | 317.1 | 99.84% | 33.28 |
| WD3615 | 11,235,162 | 90.56% | 89.71% | 292.1 | 99.84% | 34.61 |
| WD4202 | 9,973,592 | 92.09% | 91.25% | 295.9 | 99.83% | 31.4 |
| WD4204 | 11,435,114 | 90.23% | 89.42% | 281.8 | 99.84% | 35.23 |
| WD4214 | 10,245,060 | 90.77% | 90.02% | 270.1 | 99.83% | 31.43 |
| WD4232 | 10,556,834 | 91.86% | 90.94% | 398.2 | 99.83% | 32.97 |
| WD4250 | 9,394,468 | 91.79% | 91.00% | 285.3 | 99.82% | 29.41 |
| WD4301 | 8,612,124 | 91.79% | 90.95% | 310.6 | 99.84% | 27.12 |
| WD4311 | 7,786,860 | 93.52% | 92.75% | 297.2 | 99.83% | 25.23 |
| WD4322 | 10,240,062 | 91.85% | 91.06% | 288.3 | 99.83% | 32.28 |
| WD5101 | 8,968,642 | 92.02% | 91.06% | 376.4 | 99.75% | 28.31 |
| WD5114 | 11,288,668 | 91.13% | 90.18% | 290.3 | 99.77% | 35.11 |
| WD5115 | 10,597,782 | 90.78% | 89.83% | 386.3 | 99.78% | 32.92 |
| WD5123 | 7,915,768 | 91.38% | 90.39% | 378.9 | 99.74% | 25.21 |
| WD5132 | 11,742,526 | 91.78% | 90.74% | 396.1 | 99.76% | 36.95 |
| WD5134 | 10,662,638 | 92.26% | 91.41% | 359.3 | 99.75% | 33.98 |
| WD5140 | 9,335,830 | 90.49% | 89.35% | 409.9 | 99.75% | 29.01 |
| WD5203 | 10,073,824 | 91.57% | 90.76% | 299.5 | 99.83% | 31.57 |
| WD5301 | 10,002,360 | 92.92% | 92.11% | 300.1 | 99.82% | 31.98 |
| WD5321 | 8,699,886 | 91.35% | 90.41% | 382.3 | 99.74% | 27.75 |
| WD5348 | 7,279,814 | 89.92% | 88.92% | 293.2 | 99.73% | 22.95 |
| WD6101 | 10,583,484 | 91.45% | 90.39% | 287.6 | 99.83% | 32.66 |
| WD6201 | 8,695,148 | 92.81% | 91.96% | 301.5 | 99.83% | 27.61 |

**Table S4. Distribution of SNPs within different genomic regions in 26 *L. edodes* cultivars**

| **Type** | **Number** | **Percentage (%)** |
| --- | --- | --- |
| Intergenic spacer | 225,928 | 23.65 |
| Intron | 109,741 | 11.49 |
| Upstream | 124,801 | 13.06 |
| Downstream | 130,014 | 13.61 |
| 3’ UTR | 85,868 | 8.99 |
| 5’UTR | 86,276 | 9.03 |
| Shear donor | 836 | 0.09 |
| Non synonymous mutations | 94,525 | 9.90 |
| Synonymous mutations | 131,823 | 13.80 |
| Non synonymous mutations/ Synonymous mutations | 0.72 |  |
| Total | 955,236 |  |

**Table S5. Cd content of mycelium under different Cd stress**

|  | **Strain**  **name** | **Actual name（Geographical region）** | **Strain type** | **Origin** | **1**  **mg/kg** | **5 mg/kg** |
| --- | --- | --- | --- | --- | --- | --- |
| 1 | CV02 | 7402 | cultivated | China | 23.71 | 166.2 |
| 2 | CV03 | Shenxiang8 | cultivated | China | 47.63 | 296.8 |
| 3 | CV04 | shenxiang10 | cultivated | China | 31.63 | 117.5 |
| 4 | CV05 | shenxiang12 | cultivated | China | 20.25 | 100.4 |
| 5 | CV06 | L26 | cultivated | China | 39.72 | 116.1 |
| 6 | CV07 | 931 | cultivated | China | 22.17 | 88.9 |
| 7 | CV08 | Cr04 | cultivated | China | 28.54 | 100.9 |
| 8 | CV09 | CR62 | cultivated | China | 17.02 | 65.33 |
| 9 | CV101 | 3176 | cultivated | Japan | 12.43 | 59.96 |
| 10 | CV102 | 0295 | cultivated | Japan | 18.5 | 62.96 |
| 11 | CV105 | wang | cultivated | Japan | 24.42 | 74.04 |
| 12 | CV108 | X20 | cultivated | Japan | 38.17 | 56.9 |
| 13 | CV11 | Wuxiang1 | cultivated | China | 44.41 | 123.2 |
| 14 | CV12 | Xiangjiu | cultivated | China | - | - |
| 15 | CV13 | Xiangza26 | cultivated | China | 23.51 | 61.4 |
| 16 | CV14 | GX51 | cultivated | China | 25.87 | 114.7 |
| 17 | CV16 | CR02 | cultivated | China | 13.74 | 61.82 |
| 18 | CV17 | Ganxiang1 | cultivated | China | 22.63 | 86.05 |
| 19 | CV19 | Huaxiang5 | cultivated | China | 17.16 | 47.84 |
| 20 | CV20 | Huaxiang8 | cultivated | China | 31.14 | 117.4 |
| 21 | CV201 | 1151 | cultivated | China | 15.55 | 67.18 |
| 22 | CV203 | 1153 | cultivated | China | 19.25 | 47.1 |
| 23 | CV21 | Junxing8 | cultivated | China | 51.91 | 118.5 |
| 24 | CV22 | L952 | cultivated | China | 15.3 | 39.77 |
| 25 | CV24 | Minfeng1 | cultivated | China | 24.66 | 71.1 |
| 26 | CV26 | senyuan1 | cultivated | China | 21.01 | 156.6 |
| 27 | CV27 | Senyuan8404 | cultivated | China | 24.05 | 84.21 |
| 28 | CV28 | senyuan10 | cultivated | China | 24.39 | 87.22 |
| 29 | CV29 | PQ18 | cultivated | China | 32.75 | 287.1 |
| 30 | CV30 | Suxiang | cultivated | China | 23.67 | 67.8 |
| 31 | CV301 | 3161 | cultivated | China | 14.32 | 53.55 |
| 32 | CV32 | Shengxiang15 | cultivated | China | 31.35 | 178 |
| 33 | CV33 | Shengxiang16 | cultivated | China | 22.75 | 93.7 |
| 34 | CV35 | Shengxiang18 | cultivated | China | 21.75 | 102.8 |
| 35 | CV38 | Shengxiang215 | cultivated | China | 22.11 | 32.08 |
| 36 | CV39 | HuxiangF2 | cultivated | China | 28.72 | 70.28 |
| 37 | CV41 | N5 | cultivated | China | 38.47 | 120.1 |
| 38 | CV410 | 3210 | cultivated | Australia | 27.25 | 103.2 |
| 39 | CV42 | N6 | cultivated | China | 20.29 | 59.87 |
| 40 | CV43 | N7 | cultivated | China | 27.12 | 74.92 |
| 41 | CV439 | 3239 | cultivated | Australia | 26.4 | 97.01 |
| 42 | CV442 | 3242 | cultivated | Australia | 34.45 | 111.1 |
| 43 | CV443 | 3243 | cultivated | Australia | 50.53 | 133.5 |
| 44 | CV444 | 3244 | cultivated | Australia | 29.25 | 74.42 |
| 45 | CV501 | MG | cultivated | America | 23.37 | 67.12 |
| 46 | CV51 | L808 | cultivated | China | 19.55 | 53.39 |
| 47 | CV52 | J808 | cultivated | China | 18.44 | 75.37 |
| 48 | CV53 | J868 | cultivated | China | 17.4 | 122 |
| 49 | CV54 | ShouX1 | cultivated | China | 26.26 | 187.7 |
| 50 | CV55 | ZX6 | cultivated | China | 14.82 | 54.64 |
| 51 | CV57 | 0912 | cultivated | China | 36.9 | 129.7 |
| 52 | CV58 | 212 | cultivated | China | 41.04 | 91.78 |
| 53 | CV59 | 238 | cultivated | China | 49.52 | 383.5 |
| 54 | CV60 | 868 | cultivated | China | 23.2 | 27.33 |
| 55 | CV61 | Q1 | cultivated | China | 35.36 | 109.6 |
| 56 | CV65 | L135 | cultivated | China | 37.56 | 177.9 |
| 57 | CV66 | JD | cultivated | China | 38.18 | 100 |
| 58 | CV67 | 9015 | cultivated | China | 26.31 | 94.24 |
| 59 | CV68 | 241 | cultivated | China | 22.43 | 162.6 |
| 60 | CV69 | QK20 | cultivated | China | 30.14 | 93.89 |
| 61 | CV70 | 241-4 | cultivated | China | 20.38 | 148.6 |
| 62 | CV71 | 9319 | cultivated | China | 28.29 | 57.54 |
| 63 | CV72 | 939 | cultivated | China | 19.87 | 87.7 |
| 64 | CV73 | 9608 | cultivated | China | 31.55 | 44.44 |
| 65 | CV75 | XR | cultivated | China | 38.52 | 74.63 |
| 66 | WD2101 | NE China | wild | Liaoning | 18.88 | 76.14 |
| 67 | WD2102 | NE China | wild | Liaoning | 17.46 | 55.42 |
| 68 | WD2201 | NE China | wild | Jilin | 20.88 | 86.8 |
| 69 | WD2202 | NE China | wild | Jilin | 15.45 | 56.78 |
| 70 | WD2203 | NE China | wild | Jilin | 29.98 | 125.5 |
| 71 | WD2205 | NE China | wild | Jilin | 25.77 | 82.87 |
| 72 | WD2206 | NE China | wild | Jilin | 19.24 | 100.4 |
| 73 | WD2207 | NE China | wild | Jilin | 49.15 | 91.22 |
| 74 | WD3302 | Central China | wild | Zhejiang | 44.97 | 126.6 |
| 75 | WD3406 | Central China | wild | Anhui | 0.448 | 103.2 |
| 76 | WD3409 | Central China | wild | Anhui | 14.46 | 53.36 |
| 77 | WD3604 | Central China | wild | Jiangxi | 20.7 | 222.7 |
| 78 | WD3615 | Central China | wild | Jiangxi | - | 134.6 |
| 79 | WD4202 | Central China | wild | Hubei | 21.19 | 135.7 |
| 80 | WD4204 | Central China | wild | Hubei | 33.39 | - |
| 81 | WD4214 | Central China | wild | Hubei | 13.3 | 174.8 |
| 82 | WD4232 | Central China | wild | Hubei | 12.62 | 100.1 |
| 83 | WD4250 | Central China | wild | Hubei | 38.59 | 96.28 |
| 84 | WD4301 | Central China | wild | Hunan | 22.03 | 102.9 |
| 85 | WD4311 | Central China | wild | Hunan | 38.03 | 146.5 |
| 86 | WD4322 | Central China | wild | Hunan | 40.89 | 71.9 |
| 87 | WD5101 | SW China | wild | Sichuan | - | - |
| 88 | WD5114 | SW China | wild | Sichuan | 54.63 | 98.99 |
| 89 | WD5115 | SW China | wild | Sichuan | 22.55 | 70.84 |
| 90 | WD5123 | SW China | wild | Sichuan | 13.44 | 53.52 |
| 91 | WD5132 | SW China | wild | Sichuan | 10.01 | 32.76 |
| 92 | WD5134 | SW China | wild | Sichuan | 8.984 | 31 |
| 93 | WD5140 | SW China | wild | Sichuan | - | - |
| 94 | WD5203 | Central China | wild | Guizhou | 31.31 | 138.3 |
| 95 | WD5301 | SW China | wild | Yunnan | 33.58 | 189 |
| 96 | WD5321 | SW China | wild | Yunnan | 58.47 | 193.5 |
| 97 | WD5348 | SW China | wild | Yunnan | 42.81 | 208.1 |
| 98 | WD6101 | Central China | wild | Shaanxi | 32.68 | 135 |
| 99 | WD6201 | Central China | wild | Gansu | 66.39 | 147.6 |

Note:- representative hyphae pollution or does not grow, and the Cd content in the hyphae is not detected. The values are averages of three biological replicates in the footnote. NE China is the abbreviation of Northeast China, SW China is the abbreviation of Southwest China.

**Table S6. Protein-coding gene prediction in the genome.**

| **Gene set** | | **Number** | **Average gene length (bp)** | **Average CDS length (bp)** | **Average exon per gene** | **Average exon length (bp)** | **Average intron length (bp)** |
| --- | --- | --- | --- | --- | --- | --- | --- |
| De novo | GlimmerHMM | 13,151 | 2,272.6 | 1,942.6 | 3.8 | 510.55 | 117.6 |
|  | AUGUSTUS | 7,730 | 1,929.8 | 1,476.0 | 6.8 | 217.0 | 78.2 |
| Homolog | *Schizophyllum commune* | 9,286 | 1,575.7 | 912.9 | 4.6 | 197.6 | 183.1 |
|  | *Pleurotus ostreatus* | 10,620 | 1,315.7 | 897.2 | 4.4 | 203.1 | 122.4 |
|  | *Agaricus bisporus* var bisporus | 11,140 | 1,402.5 | 925.2 | 4.1 | 226.4 | 154.6 |
|  | *Coprinopsis cinerea* | 10,589 | 1,395.5 | 870.7 | 4.2 | 207.3 | 164.0 |
| Transcriptome | RNAseq | 5,411 | 3,188.8 | 1,645.3 | 8.2 | 324.8 | 73.4 |
| MAKER |  | 9,853 | 3,501.8 | 1,466.8 | 7.0 | 418.4 | 95.0 |
