# Supplementary material for: Chromosomal genome and population genetic analyses to reveal genetic architecture, breeding history and genes related to cadmium accumulation in Lentinula edodes
Source: BMC Genomics. 2022 Feb 10;23:120. doi: 10.1186/s12864-022-08325-x (PMC8832684; doi:10.1186/s12864-022-08325-x)
Supplement: Supplementary file 2 — Additional file 2. [file 12864_2022_8325_MOESM2_ESM.docx]

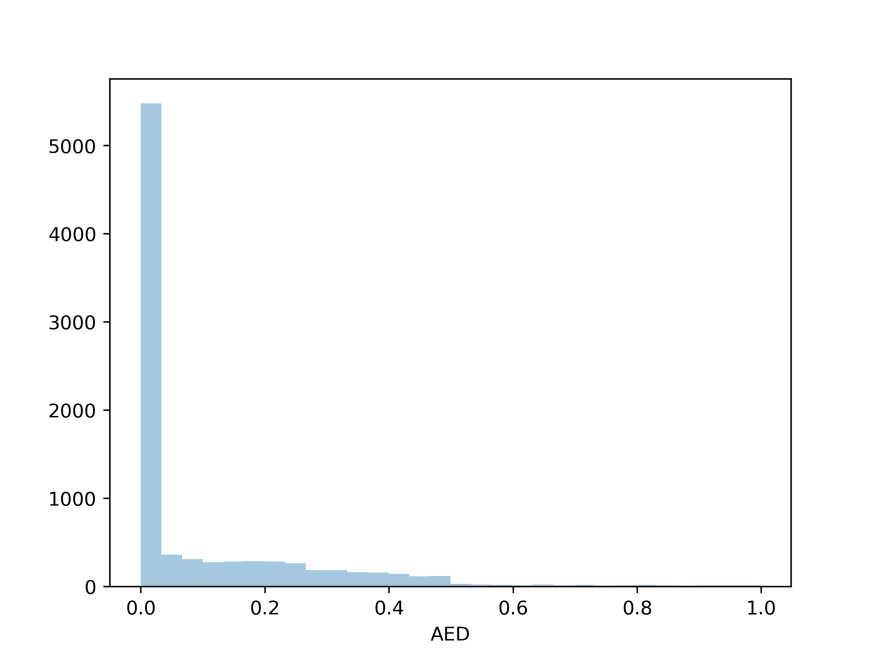


Fig. S1 The distribution of AED index by comparing gene annotation and RNA-seq data alignment.


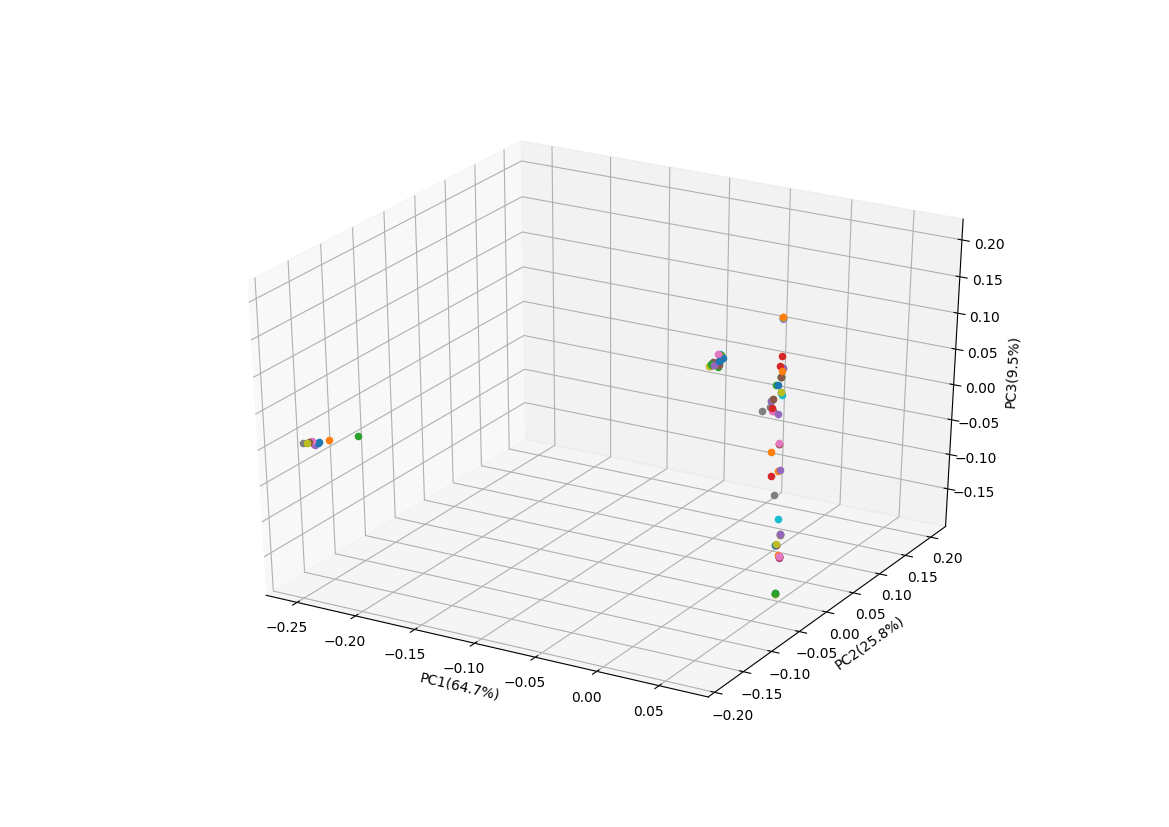


Fig. S2 Top three components in the PCA analysis for population analysis.


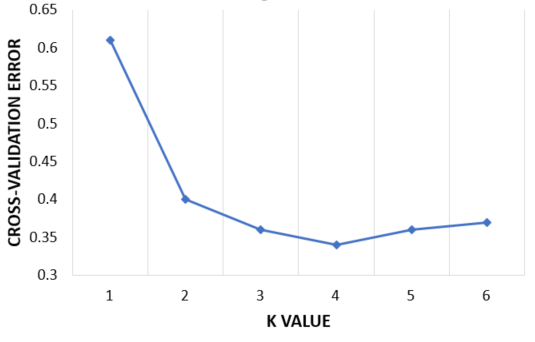


Fig. S3 Cross-validation error for population structure analysis using Admixture for different K values.


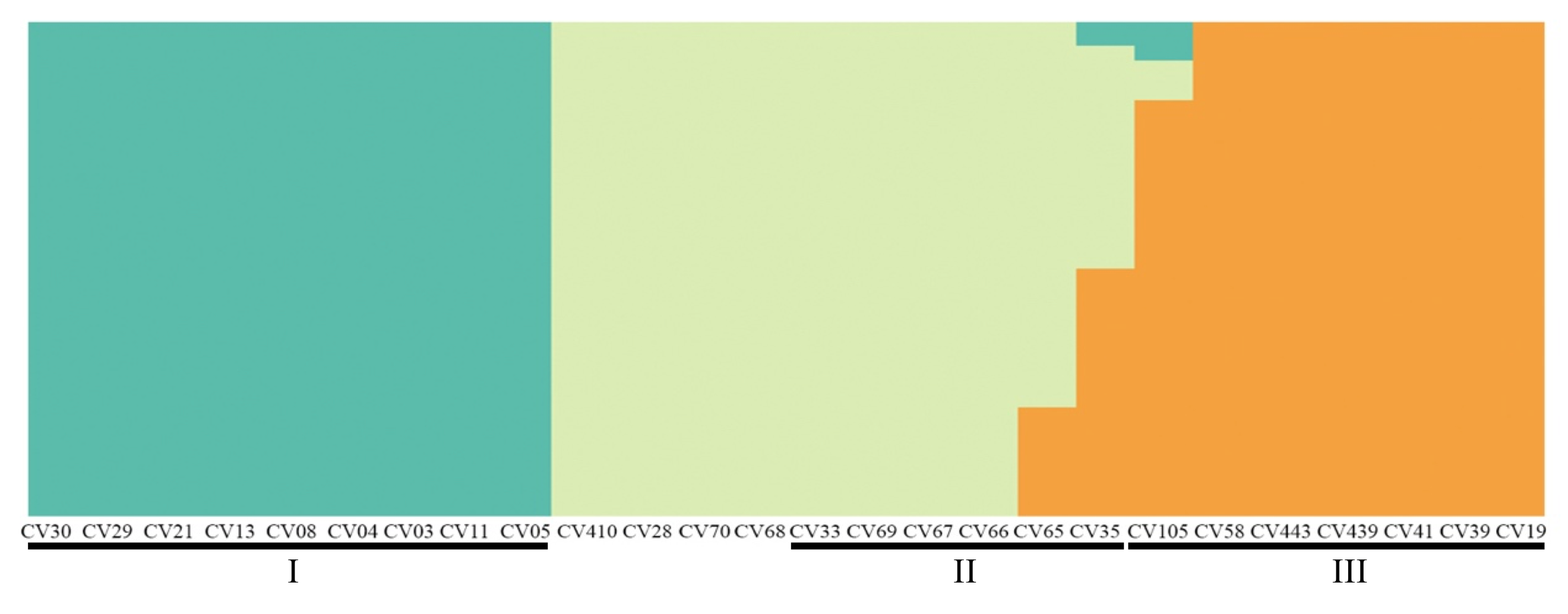


Fig. S4 Population structure analysis of 26 *L. edodes* cultivars.


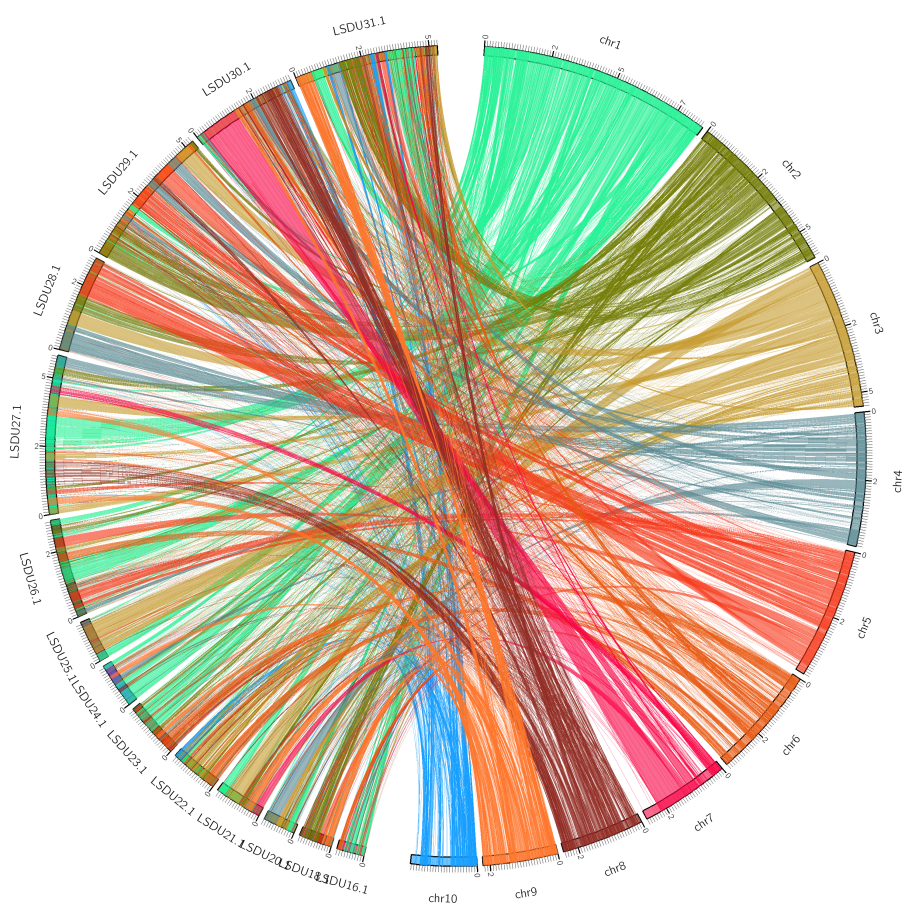


Fig. S5 Collinearity plot of B17 and L808-1 genome. Scoffold length above 1Mb.
